# Circulating Anodic Antigen (CAA): A Highly Sensitive Diagnostic Biomarker to Detect Active *Schistosoma* Infections—Improvement and Use during SCORE

**DOI:** 10.4269/ajtmh.19-0819

**Published:** 2020-05-12

**Authors:** Paul L. A. M. Corstjens, Claudia J. de Dood, Stefanie Knopp, Michelle N. Clements, Giuseppina Ortu, Irenee Umulisa, Eugene Ruberanziza, Udo Wittmann, Thomas Kariuki, Philip LoVerde, William Evan Secor, Lydia Atkins, Safari Kinung’hi, Sue Binder, Carl H. Campbell, Daniel G. Colley, Govert J. van Dam

**Affiliations:** 1 Department of Cell and Chemical Biology, Leiden University Medical Center, Leiden, Netherlands;; 2 Swiss Tropical and Public Health Institute, Basel, Switzerland;; 3 University of Basel, Basel, Switzerland;; 4 SCI Foundation, London, United Kingdom;; 5 MRC Clinical Trials Unit, University College London, London, United Kingdom;; 6 Sante Publique France, Paris, France;; 7 Malaria and Other Parasitic Diseases Division, Neglected Tropical Diseases and Other Parasitic Diseases Unit, Rwanda Biomedical Center, Ministry of Health, Kigali, Rwanda;; 8 African Leaders Malaria Alliance, Dar-es-Salam, Tanzania;; 9 Consult AG Statistical Services, Zurich, Switzerland;; 10 Institute of Primate Research, National Museums of Kenya, Nairobi, Kenya;; 11 African Academy of Sciences, Alliance for Accelerating Excellence in Science in Africa, Nairobi, Kenya;; 12 Department of Biochemistry and Structural Biology, University of Texas Health, San Antonio, Texas;; 13 Division of Parasitic Diseases and Malaria, Centers for Disease Control and Prevention, Atlanta, Georgia;; 14 Ministry of Health and Wellness, Castries, St. Lucia;; 15 Mwanza Research Centre, National Institute for Medical Research, Mwanza, Tanzania;; 16 Schistosomiasis Consortium for Operational Research and Evaluation, Center for Tropical and Emerging Global Diseases, University of Georgia, Athens, Georgia;; 17 Department of Microbiology, University of Georgia, Athens, Georgia;; 18 Department of Parasitology, Leiden University Medical Center, Leiden, Netherlands

## Abstract

The Schistosomiasis Consortium for Operational Research and Evaluation (SCORE) was funded in 2008 to conduct research that would support country schistosomiasis control programs. As schistosomiasis prevalence decreases in many places and elimination is increasingly within reach, a sensitive and specific test to detect infection with *Schistosoma mansoni* and *Schistosoma haematobium* has become a pressing need. After obtaining broad input, SCORE supported Leiden University Medical Center (LUMC) to modify the serum-based antigen assay for use with urine, simplify the assay, and improve its sensitivity. The urine assay eventually contributed to several of the larger SCORE studies. For example, in Zanzibar, we demonstrated that urine filtration, the standard parasite egg detection diagnostic test for *S. haematobium*, greatly underestimated prevalence in low-prevalence settings. In Burundi and Rwanda, the circulating anodic antigen (CAA) assay provided critical information about the limitations of the stool-based Kato–Katz parasite egg-detection assay for *S. mansoni* in low-prevalence settings. Other SCORE-supported CAA work demonstrated that frozen, banked urine specimens yielded similar results to fresh ones; pooling of specimens may be a useful, cost-effective approach for surveillance in some settings; and the assay can be performed in local laboratories equipped with adequate centrifuge capacity. These improvements in the assay continue to be of use to researchers around the world. However, additional work will be needed if widespread dissemination of the CAA assay is to occur, for example, by building capacity in places besides LUMC and commercialization of the assay. Here, we review the evolution of the CAA assay format during the SCORE period with emphasis on urine-based applications.

## INTRODUCTION

The Schistosomiasis Consortium for Operational Research and Evaluation (SCORE) was funded in 2008 to conduct research to support country programs to control and eliminate schistosomiasis.^1^ A high priority was to support work on improved diagnostic assays. SCORE’s work on such tools for the mapping of schistosomiasis is described in another article in this supplement.^2^ In addition to better mapping diagnostic assays, developing a highly sensitive and specific test for detection of infection with *Schistosoma mansoni* and *Schistosoma haematobium* has also been a high priority*.* Such an assay is likely to increase in importance as prevalence and intensity continue to decrease in many places and elimination is within reach.

In 2009, SCORE held a meeting to help define which of the many opportunities for investment in diagnostics would be most likely to yield concrete results that would be of use to control and elimination programs within the SCORE time frame.^2^ Participants included individuals working with parasite diagnostics and those using cutting-edge technologies for other detection purposes. Discussions covered a range of approaches–including nucleic acid, antigen, and antibody tests. It was decided that SCORE would provide support for the further development of a laboratory-based test, the upconverting particle-lateral flow circulating anodic antigen (UCP-LF CAA) assay, developed and housed at the Leiden University Medical Center (LUMC). Priority goals included improved sensitivity of the test, potentially for detection of a single worm; modifications that might increase its accessibility and usability for programmatic decision-making; and evaluation of its performance in real-world settings. The envisioned uses of the UCP-LF CAA assay were wide-ranging and included assessing the results of field mapping and surveillance tools, measuring prevalence and intensity in settings approaching elimination, and determining the effectiveness of praziquantel treatment.

## CIRCULATING ANODIC ANTIGEN

The UCP-LF CAA assay detects a *Schistosoma* genus-specific, adult worm gut-derived antigen. Circulating anodic antigen (CAA) is a glycosaminoglycan-like molecule that is regurgitated into the host’s bloodstream.^3,4^ This antigen is distinct from the circulating cathodic antigen (CCA) used in the point-of-care (POC) CCA mapping tool for *S. mansoni.*^2^

CAA’s polysaccharide structure renders it very stable, and it has not been identified in organisms other than schistosomes. CAA has a strong negative charge and is at least 10 kDa, but can vary in molecular weight.^3^ The concentration of this antigen circulating in the bloodstream is believed to correlate with the infection burden of schistosomes in the host,^5,6^ although the relationship between worm burden and CAA levels may differ between *Schistosoma* species. CAA is cleared rapidly, so detection of CAA in the bloodstream indicates active infection. Rapid decrease in the serum level of CAA after treatment with praziquantel, sometimes within several hours, has been documented.^7^

Early diagnostic assays for CAA used mouse monoclonal anti-CAA antibodies in a quantitative serum-based ELISA test.^5,6,8,9^ The development of a lateral flow (LF) test platform, combined with a unique and highly sensitive luminescent reporter label—upconverting particles (UCP)^10^—represented a major improvement in sensitivity. The UCP reporter is a unique background-free label that is detected and quantified upon excitation with low-energy infrared light. The UCP-LF CAA test improved the lower limit of detection (LLOD) more than 10-fold as compared with the ELISA.^11^

When moving to larger batch production of test materials for third party use, the quality control (QC) threshold for this UCP-LF CAA assay was set to 10 pg/mL for use with a wet UCP reporter conjugate.^3^ The first clinical study describing successful use of this UCP-LF CAA test in an endemic setting (with test materials supported by SCORE) was on serum samples from a Tanzanian cohort from rural villages close to Lake Victoria with high rates of *S. mansoni* and HIV.^12^

## SERUM ASSAY IMPROVEMENTS

### Simplifying the serum test.

SCORE resources allowed a speedy implementation of several modifications that made the UCP-LF CAA assay more user-friendly. A dry reagent format was established that allowed storage and worldwide, cold, chain-free shipping of reagents.^13^ The use of dry reagents also meant that the intricate step of sonicating the wet UCP reporter conjugate before mixing it with the clinical sample was no longer needed. Performing the dry format test only requires a standard microtube centrifuge, a shaker, and manual pipettes, along with a lightweight, portable LF strip reader for the analysis. As initially developed, a negative aspect of the dry reagent format was an increase in the QC threshold to 30 pg/mL, versus 10 pg/mL using the wet assay format.^3^

The UCP-LF CAA serum test was evaluated in the laboratory over a period of 18 months by the Department of Serology of the Ampath Laboratories in South Africa. Results obtained from 2,304 samples showed excellent performance (outperforming the CAA ELISA) and indicated that the 30 pg/mL positivity threshold was robust enough to handle batch-to-batch production lot variability.^13^ In research settings within a single production batch, a lower threshold could be considered by including an appropriate set of negative controls and standards. However, for clinical use, predetermined, non-flexible thresholds need to be set that cannot be below the QC levels used during production.

### Increasing sensitivity of the serum test.

Although these formats of the UCP-LF CCA assay allowed for the detection of low-level infections in some travelers,^11^ their maintenance at QC thresholds of 10–30 pg/mL was not sufficient to identify all cases of infection, especially those with very low worm burdens. SCORE therefore provided support to accelerate ongoing studies at LUMC to further increase the sensitivity of this assay.

Past literature using ELISA-based testing suggested that a fit, single worm pair would result in a CAA level of 3–8 pg/mL in serum.^5,14^ This concentration was derived from in vitro worm culture studies and from baboons experimentally infected with *S. mansoni.* These studies indicated an average production of about 40 ng CAA per worm pair per day. However, more recent clinical studies using the more sensitive UCP-LF CAA test indicated active infections in travelers with CAA serum levels even less than 1 pg/mL.^11,15^ We speculate that when infection intensities are very low, variation in CAA levels may be observed; circadian rhythms and feeding patterns as well as immune-mediated clearance mechanisms of the host may play a role. Current thinking is that a single worm pair might produce a minimum CAA level of 1 pg/mL serum (unpublished). However, single sex and immature worms as well as worms recovering after praziquantel (PZQ) drug treatment may produce less CAA than a healthy egg-producing worm pair [unpublished], as demonstrated recently in a controlled human *Schistosoma* infection study with single sex (male) cercariae at LUMC.^16,17^

Because LF assays are limited in terms of sample volume that can be applied to the cassette, increasing the sensitivity of the UCP-LF CAA assay required the addition of a concentration step. The UCP-LF CAA test includes an extraction step with trichloroacetic acid (TCA), which leaves carbohydrate structures such as CAA in the clear supernatant fluid while precipitating proteinaceous material. In the standard assay, 20 μL of the supernatant fluid (containing 2% w/v TCA) is 5-fold diluted with assay buffer, and this 100 μL can be applied to the LF strip. To enhance sensitivity, the TCA supernatant fluid can be concentrated using Millipore Amicon centrifugal filtration devices (Merck Chemicals B.V., Amsterdam, The Netherlands) with a 10-kDa molecular weight cutoff. With this approach, the QC thresholds were improved to 1 pg/mL with the wet format and 3 pg/mL for the dry format.^3^ This assay is referred to as “SCAA500,” with “S” indicating serum and “500” the equivalent amount of serum (in μL) analyzed on the LF strip. Application of the SCAA500 test improved detection of low-level infections in travelers^15^ and substantially improved detection sensitivity in low endemicity settings.^18–22^

## MODIFYING THE TEST FOR USE WITH URINE

Clearance of CAA from the bloodstream occurs at least partly via the kidneys,^23,24^ suggesting urine specimens could potentially be used for assessing infection status. The CAA-ELISA test yielded low sensitivity when used on urine samples compared with serum, in part due to lower CAA concentrations in urine.^25^ By contrast, the UCP-LF CAA assay format as used for serum analysis did accommodate the testing of urine for CAA, with similar analytical sensitivity.

There are several major advantages to testing urine rather than serum. Because CAA is stable, urine samples do not need a cold chain for several days when transported from the field to the laboratory. Furthermore, urine collection is noninvasive and does not require trained medical staff, and larger volumes can be obtained. However, as CAA concentrations in urine are generally at least 10-fold lower than that in blood, sample concentration is needed for successful detection of low-level infections. Fortunately, unlike with serum, the TCA extraction step forms only minor precipitates, and TCA supernatant fluid from urine samples allows virtually infinite concentration as the viscosity does not increase substantially upon concentration.^26^ The urine test can be performed on fresh or frozen urine, although centrifugation before TCA extraction can lead to loss of CAA, especially in turbid urines or after freezing (Corstjens and de Dood, unpublished). The ability to concentrate specimens ensures a good LLOD for testing individual samples. It may also allow for pooling of samples from multiple people, which might provide a more cost-effective way to assess infection status in populations.^27^

The urine assay performed with a 4-mL filtration device is referred to as “UCAA2000,” with “U” indicating urine and “2000” the amount of urine (in μL) analyzed on the LF strip.^3^ Several formats of the UCP-LF CAA assay specifically for urine analysis are available and are shown in Table 1.^3^ One modification relates to the capacity of a given concentration device. The largest centrifugal devices (Amicon ultra centrifugal filters) can hold up to 15 mL TCA supernatant. Other devices requiring gas pressure (Amicon stirred cell devices) can hold up to 400 mL. A second format uses a stock solution with a higher concentration of TCA (12% w/v rather than 4% w/v). This modification (*hT* in Table 1) improves analytical sensitivity and lowers the limit of detection by an additional 30–40%. Depending on the sensitivity needed, the use of a higher TCA concentration may allow for testing with a smaller concentration device, thus decreasing cost and sample preparation time. Some care needs to be taken in case of urolithiasis: reaction of TCA with calcium oxalate or calcium phosphate precipitates in the urine can lead to unexpected gas formation on mixing.

**Table 1:** Detection limit (quality control [QC] threshold) of the UCP-LF CAA assay formats

Assay format*	Amicon device†	TCA (w/v) (%)‡	Threshold (pg/mL)§
SCAA20	None	4	30/10
SCAA500	0.5 mL	4	3/1
UCAA10	None	4	30/10
UCAA250	0.5 mL	4	3/1
UCAA2000	4 mL	4	0.3/0.1
UCAA7500	15 mL	4	1/0.03
UCAA20000	40 mL	4	0.03/0.01
UCAA***hT***17	None	12	20/6
UCAA***hT***417	0.5 mL	12	2/0.6
UCAA***hT***3333	4 mL	12	0.2/0.06
UCAA***hT***12500	15 mL	12	0.06/0.02
UCAA***hT***33333	40 mL	12	0.02/0.005

TCA = trichloroacetic acid.

* The sample matrix is indicated by S for serum and U for urine. *hT* indicates that the sample is extracted with ⅙ volume of 12% w/v TCA. Otherwise, extraction is with equal volumes of 4% TCA. The end concentration of the TCA supernatant is always 2% w/v TCA. The equivalent of the volume of the original clinical sample analyzed on the strip is indicated in μL (the number after CAA or CAA***hT***).

† Amicon centrifugal devices are available in 0.5, 4, and 15 mL. The 40-mL device is a stirred cell device with an identical 10-kDa Amicon filter membrane as that used in the centrifugal devices. Stirred cell devices are operated by pressure.

‡ TCA solution used for extraction of the clinical sample.

§ QC threshold for the assay using freshly sonicated wet conjugate or dry reagents, respectively.

With respect to clinical sensitivity, the SCAA20 performs comparably to the UCAA250^28^ and the SCAA500 compares with the UCAA2000 assay.^19,21^ The latter two assays approach the sensitivity needed to possibly detect infection with a single worm or worm pair. Whether to test serum or urine is, in part, dependent on the circumstances. Whereas collection of urine is simpler and less invasive, testing urine requires a more elaborate concentration step because of lower CAA levels in urine. Analytically, the UCAA2000 assay detects 10-fold lower concentrations of CAA in urine than the SCAA500 in serum. The 10-fold better analytical sensitivity requires only a 4-fold larger sample volume. This is because there is much less precipitate after TCA extraction and generally much lower or no background when using concentrated urine samples.

### Use of the urine assay to evaluate graded infections in baboons.

**Figure 1.**
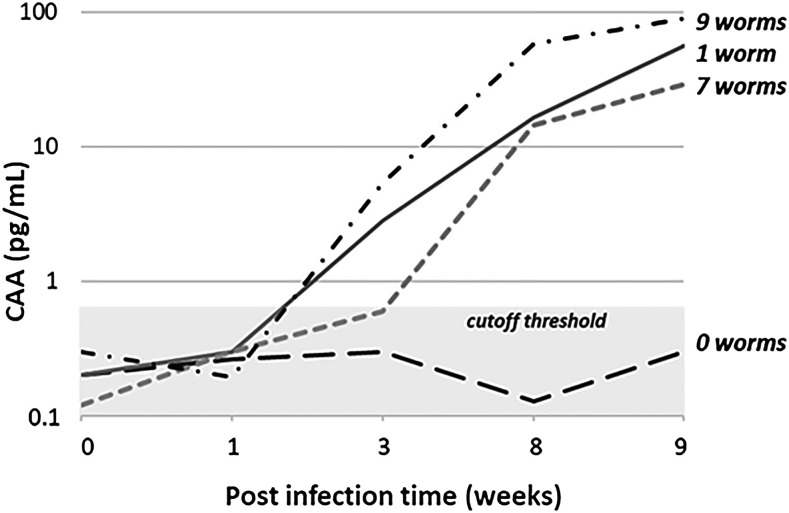
Circulating anodic antigen (CAA) levels detectable in urine specimens collected at baseline, 1, 3, 8, and 9 weeks after infection from baboons infected with *Schistosoma mansoni* cercariae and perfused at week 9 to determine worm burdens. CAA levels, shown on the *y* axis on a logarithmic scale, were measured in urine with the UCAA250 assay and demonstrate detectable levels (above the UCAA cutoff threshold, gray area) by week 3.

Once urine testing was established, a retrospective study was undertaken to test for CAA in the stored (−20°C) urines from a baboon infection study conducted previously at the (IPR, Nairobi, Kenya). Four of the baboons infected (5–25 *S. mansoni* cercariae/baboon) had urine specimens collected at baseline and 1, 3, 8, and 9 weeks after infection (Figure 1). These baboons were perfused at 9 weeks of infection to determine their worm burdens and found to have 0, 1, 7, or 9 adult schistosome worms.

In a study supported by SCORE, the baboon urines were thawed and analyzed for CAA. As seen in Figure 1, the baboon with 0 worms on perfusion did not have detectable levels of CAA in its urine, whereas baboons with 1, 7, and 9 adult worms showed detectable levels of CAA by the UCAA500 assay by 3 weeks postinfection. Although there was no clear distinction between CAA levels based on the number of detected worms in this analysis, a larger study that included these baboons did show an obvious relationship between the number of cercariae used for infection and CAA levels (personal observation). Baboons are natural hosts for *S. mansoni*, and the ability of this assay to detect a baboon with one adult worm suggests that the assay is able to detect extremely low-level human infections.

## SCORE STUDIES APPLYING THE UCAA2000 ASSAY

The UCAA2000 assay format was used in several studies designed or co-funded by SCORE, mainly to evaluate field performance of the POC-CCA urine test and also to investigate prevalence by UCP-LF CAA assays in relation to a standard parasitological assay. Many of these studies were implemented in the context of SCORE’s major field studies, for example, on how best to carry out mass drug administration^29^ and on programmatic approaches that might be useful in elimination programs.^30^ In addition, with in-country partners, SCORE undertook extensive mapping exercises in endemic areas with high to very low levels of prevalence. These studies usually relied on the POC-CCA assay and Kato–Katz parasitological assays for detection of *S. mansoni* infections and the parasitological urine filtration assay for detection of *S. haematobium* infections. In several of these studies, the UCP-LF CAA assay was used as a confirmatory test for the POC-CCA and Kato–Katz or urine filtration results. The following summarized studies benefited from this approach.

### Zanzibar–urogenital schistosomiasis (*S. haematobium*).

The Zanzibar Elimination of Schistosomiasis Transmission (ZEST) program is a multi-partner effort to eliminate schistosomiasis from Unguja and Pemba islands of the Zanzibar archipelago. SCORE funded a cluster-randomized trial, the Zanzibar Elimination Study that was embedded within ZEST, which ran from 2011 to 2017.^30–33^ The study comparatively assessed the impact of biannual mass drug administration plus additional interventions over 5 years. It included annual cross-sectional surveys of *S. haematobium* prevalence and intensity of infection in schoolchildren and adults, measured by urine filtration.

In 2013, the urine-based UCP-LF CAA assay was established in the Public Health Laboratory Ivo de Carneri on Pemba. Local staff, trained by LUMC experts, used the UCP-LF CAA assay to evaluate 1,200 urine samples from the 2013 cross-sectional survey in schoolchildren who had also been tested with urine filtration.

**Figure 2.**
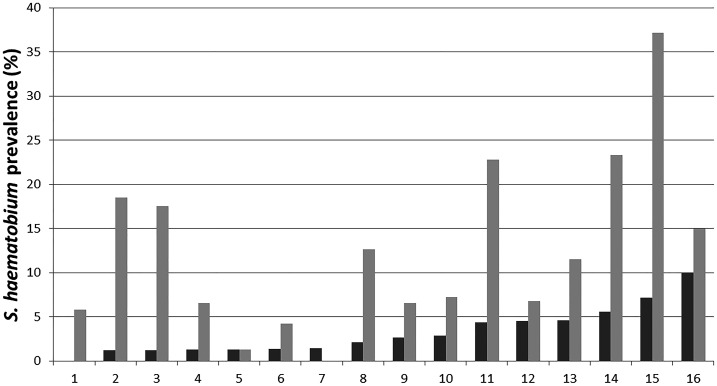
Prevalence of *Schistosoma haematobium* was determined by the standard filtration of 10 mL urine (dark bars) and the UCAA2000 (light gray bars) applied to the same urine samples collected from children attending 16 schools on Pemba island. Figure redrawn, based on Knopp et al.^35^

This study was the first time that the performance of the high-sensitivity UCAA2000 assay, which used 2 mL of urine, was evaluated for use in a potential schistosomiasis elimination setting and a local laboratory.^34^ The evaluation found that a large number of specimens thought to be negative for *S. haematobium* infections by urine filtration were found to be positive using this UCP-LF CAA assay (Figure 2). Hence, using the standard parasitologic method underestimated the *S. haematobium* prevalence in Zanzibar. Urine filtration identified 41 (3.1%) of the samples as egg positive, whereas the CAA2000 assay with trace considered as negative indicated that at least 159 (13.3%) of the individuals were actively infected.

### Burundi–intestinal schistosomiasis (*S. mansoni*).

Burundi had been conducting mass drug administration against schistosomiasis for many years. Based on Kato–Katz testing in sentinel sites, the prevalence was believed to be quite low. In 2014, in a study undertaken by the Ministry of Health of Burundi, the Ending Neglected Diseases (END) Fund (https://end.org), the Schistosomiasis Control Initiative (SCI Foundation; https://schistosomiasiscontrolinitiative.org), and SCORE, the overall prevalence in 17,331 children tested by POC-CCA with trace results considered as positive was 42.8%. In a subsample of 8,842 of these children, Kato–Katz testing yielded a prevalence of 1.5% and POC-CCA testing with trace considered as positive yielded a prevalence of 41.3%.

The investigators selected 398 urine specimens from children with both Kato–Katz and POC-CCA results from eight schools with different levels of prevalence. The Kato–Katz and POC-CCA (trace considered as positive) prevalence on these 398 specimens was 6.8% and 53.5%, respectively. When these 398 urine specimens were tested by the UCAA2000 assay at LUMC, the prevalence was 46.5%.^35^ Latent Class Analysis (LCA) demonstrated that approximately half of the 129 trace readings of the POC-CCA assay positives were true positives. In this study, the UCAA2000 assay identified nearly 7-fold more infected individuals than Kato–Katz, demonstrating the need for better field-usable diagnostics.

### Rwanda–intestinal schistosomiasis (*S. mansoni*).

The Ministry of Health of Rwanda in conjunction with the same partners as in the Burundi survey conducted a similar mapping project in Rwanda. When traces were considered positive, the POC-CCA results from 19,371 children showed a prevalence of 36.1%. In 8,697 of these children tested with both Kato–Katz and POC-CCA, a prevalence of 2.0% and 37.5%, respectively, was revealed.

Subsequently, as in Burundi, selected urine specimens that had been tested by POC-CCA as part of the survey were sent to LUMC for CAA testing. The 396 specimens were selected from eight schools that represented a spectrum of prevalence levels. By Kato–Katz and POC-CCA, 8.1% and 65.7% among these 396 specimens were positive, respectively, and 136 (34.3%) of the readings were traces in POC-CCA. By the UCAA2000 assay, the prevalence was 44.2%. Latent class analysis indicated that the POC-CCA assay has much higher sensitivity and specificity than does the Kato–Katz assay.^36^ Based on the UCAA2000 results and comparing all assays using LCA, approximately 50% of POC-CCA trace positives were estimated to be true positives.

### St. Lucia–intestinal schistosomiasis (*S. mansoni*).

The island nation of St. Lucia was highly endemic for *S. mansoni* at one time, with prevalence levels in the 60% range and intensities of infection comparable to those in Africa.^37^ With a strong research and control effort in the late 1960s to the early 1980s, followed by considerable development and expansion of the public health infrastructure across the country, it was believed that transmission of *S. mansoni* might be negligible or eliminated. In 2017, the Department of Health and Wellness of St. Lucia partnered with the Pan American Health Organization, the Centers for Diseases Control and Prevention, and SCORE to survey about 1,487 children aged 8–11 years, selected from all 63 public primary schools on St. Lucia, using the POC-CCA urine assay, a soluble *S. mansoni* egg antigen ELISA test, and Mansoni Adult Microsomal Antigen (MAMA) immunoblot assays from dried blood spots. In addition, urine specimens from some of the children who had inconsistent test results were sent to LUMC for evaluation by the UCAA2000 assay. Some samples had very low positive or equivocal results by each of the assays, but these were not necessarily in the same children. On retesting with the same assays, none of the specimens were confirmed positive. From these results, it is clear that if *S. mansoni* infections still exist on St. Lucia, they are not present in the children who were surveyed.^38^ The finding of some inconsistent low-level positive results by the various assays indicates they are likely false positives, which highlights the difficulties in setting assay thresholds with 100% accuracy when targeting maximal sensitivity of any diagnostic test.

### Tanzania–intestinal schistosomiasis (*S. mansoni*).

During the final survey year of the SCORE gaining control study,^30^ a subset of 216 urine specimens from 9- to 12-year-old schoolchildren from 16 schools were sent to LUMC for examination by the UCP-LF CAA assay. These specimens had been tested in Tanzania by the POC-CCA assay, and stools from these 216 children had also been tested by the Kato–Katz assay. The POC-CCA results detected a prevalence of *S. mansoni* infection of 92.1% (of which 52.3% were traces), whereas Kato–Katz testing resulted in a prevalence of 8.8%. When the 216 specimens were assayed at LUMC by the UCAA2000 assay, the prevalence was 70.8%. Thus, confirmatory testing by the UCP-LF CAA assay again indicated that prevalence was much more than indicated by Kato–Katz, and there was a much better, albeit not exact, correlation between the UCP-LF CAA and the POC-CCA assay. Of the 19 low-intensity Kato–Katz positives (mean eggs/gram feces = 68) in this group, six (31.6%) were negative by both UCP-LF CAA and POC-CCA. This apparent discrepancy, because the presence of eggs in stool indicates active infection, has not been adequately resolved.

## OTHER STUDIES DURING SCORE WHERE THE CAA URINE ASSAY WAS APPLIED

**Figure 3.**
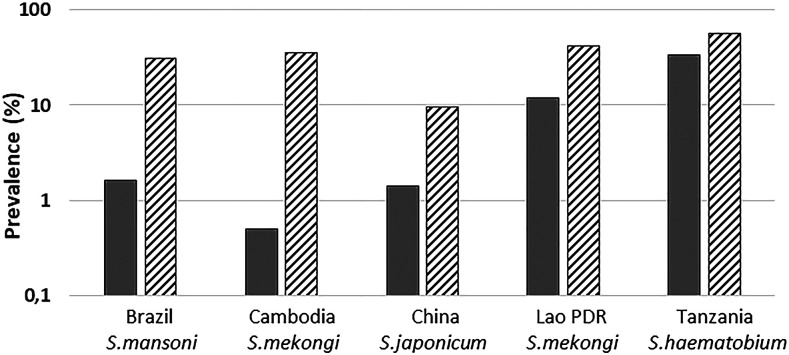
Comparative prevalence levels shown on the *y* axis on a logarithmic scale in five different countries based on egg counts (urine or stool) and the UCAA2000 assay for antigen detection. Areas with different endemicity settings: low endemicity settings in Brazil (*n* = 258),^19^ Cambodia (*n* = 196),^22^ and China (*n* = 317)^20^; medium- through high-endemicity settings in Lao PDR (*n* = 181)^22^ and Tanzania (*n* = 44).^29^ Solid bars indicate prevalence based on egg microscopy in urine or stool (species depending). Hatched bars indicate prevalence by CAA as determined in urine with the UCAA2000 assay.

It seemed clear that multiple other field-based studies could benefit from the advancements in the high-sensitivity urine-based CAA assay. Countries where the assay has been used include Brazil,^19^ Cambodia and Lao People’s Democratic Republic,^22^ People’s Republic of China,^20^ and Tanzania.^29^ These studies mainly focused on endemic settings where prevalence and intensity of infection (whether with *S. mansoni*, *S. haematobium*, *Schistosoma japonicum*, or *Schistosoma mekongi*) was very low or transmission was thought to have been interrupted. The studies sought to determine the usefulness of the UCAA2000 assay at its current levels of sensitivity and specificity for detecting low-level infections, as well as its ability to evaluate cure rates.^7^ As predicted,^39^ prevalence levels found in these studies by the UCAA2000 assay were dramatically higher (Figure 3) than those estimated based on egg counts in urine or stool.

## CONCLUSIONS AND FUTURE POSSIBILITIES

The SCORE investment contributed substantially to enhancement of the UCP-LF CAA format for serum, implementation of a sample concentration step, development of protocols for using dry reagents, and demonstration that CAA can be sensitively detected in urine. During the SCORE operational research period, studies in multiple settings demonstrated that the UCP-LF CAA assay is a highly sensitive and specific tool to determine prevalence of active infection at all levels, indirectly estimate worm burdens, and evaluate treatment programs. In addition to essential support of the laboratory advances in the development of the UCP-LF CAA assay, SCORE was involved in first efforts to examine the potential of commercialization of this assay, which resulted in a collaboration between LUMC and PATH (Seattle, Washington) in which PATH, as an intermediary for the BMGF, performed an official visit to LUMC to validate assay claims. This resulted in a first collaboration with a leading U.S. diagnostic company to evaluate the possibility of implementing the CAA assays in a commercially available diagnostic POC platform. Similar activities are currently ongoing with FIND (Geneva, Switzerland). Moreover, a number of efforts have yielded multiple, comprehensive Target Product Profiles,^40^ but these have not yet led to the much needed commercialization of a POC test.

There is a great public health need for the assay, now and in the future. Its current use is constrained by cost and the limited capacity of LUMC to run large numbers of samples. Were the current CAA assay to be available in a POC format, it would address the high-priority need for a field-applicable, sensitive, and specific assay for *S. haematobium* and might be an improvement over the available field assays for *S. mansoni.* It would also allow for programmatic implementation of test-and-treat approaches and allow for efficient post-elimination surveillance. SCORE supported a small effort by the manufacturer of the POC-CCA assay and LUMC to develop a POC-CAA assay. Several prototypes were made and evaluated in a laboratory in Tanzania and using specimens from the SCORE Elimination Study on Zanzibar, as well as other stored specimens, but these prototypes did not meet minimal expectations for sensitivity. It is hoped that continued efforts including by companies involved in the development of diagnostics, as indicated earlier, will continue and soon lead to commercially available CAA tests.

Further debate may focus on what sensitivity (in terms of CAA level) is needed to appraise whether elimination of transmission has occurred, in other words, that individuals or risk groups in an area are unlikely to be contributing to transmission. Should this be the lowest detectable level achievable or just below the level of 1 pg/mL (CAA blood level) currently assumed to be the lowest level that might still indicate the presence of a patent worm pair? For the coming decade(s), surveillance for active infections will remain necessary in areas with very, very low prevalence and in those where interruption of transmission has been achieved and interventions stopped. This surveillance will be critical both to respond quickly to re-emergence of infections and to verify elimination. As the criteria for establishing elimination are defined and established and country-level elimination begins to be verified, people in those countries with active infections may occur sporadically and surveillance will be essential. At that point, it is likely that simpler and less expensive antibody-based assays will be used to determine exposure in those individuals. These assays should also be suitable for testing young populations (i.e., individuals born after verification of the interruption of transmission) for surveillance.

Rapid antibody assays detecting the humoral response against egg antigens and cercariae using the UCP-LF platform were also developed during the SCORE initiative.^3^ Such assays, once they are evaluated, standardized, and commercialized, will also clearly have a role in case-by-case detection in maternal–child health settings, primary care facilities, and traveler’s clinics in non-endemic areas.

There are several ongoing and potential future research uses for the UCP-LF CAA assay. These include 1) monitoring of controlled human schistosome infection trials^41,42^; 2) accurate assessment of the efficacy of vaccines in clinical trials^43^; 3) providing sensitive, confirmatory testing on subsamples from population-based studies; 4) determining the efficacy of drug treatment and the presence of “recovering” worms, either in an endemic setting or for the diagnosis of imported schistosomiasis in the context of travel medicine; 5) detection of CAA in dried blood spots, which would allow stable storage and collection of blood by finger prick, and could allow the use of a single specimen for multiple diagnostic purposes^44,45^; and 6) continuing its current use of estimating the gap between prevalence by mapping surveys and the estimated levels of infection as determined by LCA and other mathematical modeling methods.

## CONCLUSIONS AND FUTURE POSSIBILITIES

Having the UCP-LF CAA assay or a variation of this assay more available is of high priority. Although it would be useful for diagnosing individual infection status in travelers and others from developed countries spending time in endemic areas, the primary need is for schistosomiasis control and elimination programs, especially as prevalence in many endemic areas decreases and elimination becomes a more realistic programmatic goal.^46^ Whether through commercialization of the assay or public/private efforts, further exploration of how to build global capacity to implement this critical tool is essential.
